# Drain fluid amylase as a predictor of postoperative salivary fistula in cases with benign parotid tumours

**DOI:** 10.1186/s12903-020-01166-8

**Published:** 2020-07-02

**Authors:** Yusheng Lu, Shijian Zhang, Canbang Peng, Wenyi Yang, Chenping Zhang, Zhenhu Ren

**Affiliations:** 1 Department of Oral and Maxillofacial Surgery (Zhang Zhiyuan Academician Workstation), Hainan Western Central Hospital (Shanghai Ninth People’s Hospital), Hainan Branch, Danzhou, Hainan 571700 China; 2 Department of Oral and Maxillofacial-Head and Neck Oncology, Ninth People’s Hospital, School of Medicine, Shanghai Jiao Tong University, 639 Zhi-zao-ju Road, Shanghai, 200011 China

**Keywords:** Drain fluid amylase, Benign parotid tumour, Surgery, Postoperative salivary fistula, Predictor

## Abstract

**Background:**

Late diagnosis of a salivary fistula increases the risk of wound infection and scarring. The purpose of the present study was to identify a quantitative predictor of postoperative salivary fistula for cases treated with surgery.

**Methods:**

Demographic, intraoperative and postoperative parameters for 57 cases that received surgery for benign parotid tumours were recorded from June 2017 to June 2018, of which 18 cases developed salivary fistulas. These data were analysed using univariate and binary logistic regression analyses as well as receiver operating curve analysis.

**Results:**

Drain fluid amylase concentration was positively correlated with salivary fistula development (*p* <  0.001), with an odds ratio of 1.14 for a 1 KU/L increase in concentration and an optimal receiver operating curve cut-off value of 51,100 U/L for predicting salivary fistula development. Cases wherein the parotid–masseteric fascia remained intact were associated with a lower risk of salivary fistula development (*p* = 0.006).

**Conclusion:**

Drain fluid amylase concentration may be a valuable predictor of postoperative salivary fistula in cases with benign parotid tumours.

## Background

Salivary gland neoplasms account for a small proportion of all tumours of the head and neck, but they widely vary in type and exhibit remarkable variation in clinical and pathological manifestations [1]. Overall, benign salivary gland tumours accounted for the majority and most occur in the parotid [2]. Surgery is an accepted conventional therapy for benign salivary neoplasms, with parotidectomy being the most common. However, the complications including facial nerve paresis or paralysis as well as others such as salivary fistulas and Frey’s syndrome that significantly affect the quality of life of patients should be taken into consideration. If a salivary fistula is not diagnosed sufficiently early, there is an increased risk of wound infection and visible scarring. A salivary fistula may trigger a self-limiting swelling of the face; therefore, postoperative management often involves applying pressure dressing to the parotid region for several days. However, significant side effects of this include constant discomfort, inconvenience while eating and talking and poor aesthetic appearance, and patients may suffer from malaise engendered by pressure to their head and face. To reduce unnecessary discomfort, clinicians need an appraisal system that predicts whether a patient would need preventive management or increased attention to monitor for salivary fistula development.

The purpose of the present study was to establish a quantitative predictor for postoperative salivary fistula development in patients undergoing parotidectomy for benign parotid tumours. Such a predictor could be used to evaluate whether a patient requires measures such as the application of a pressure dressing and administration of anticholinergics [3] for the prevention and/or early treatment of a salivary fistula.

## Methods

During the period from June 2017 to June 2018, we followed 57 cases of surgically resectable benign tumours in the parotid region treated with parotidectomy at our department. Two patients exhibited bilateral tumours; each side was considered an independent case. Four experienced surgeons performed the surgical procedures, which included tumour enucleation, tumour and partial superficial parotidectomy without nerve dissection and tumour and partial superficial parotidectomy with the dissection of the main trunk or branches of the facial nerve. Each procedure was carefully selected according to the preoperative diagnosis as well as the size, texture and location of the tumour. And enucleation was only applied to cases diagnosed as warthin tumour before surgery.

At the end of the operation, a negative pressure drainage device was placed in the operative region to release postoperative secretion. The restranining bandage was applied immediately after surgery, and switched to a medical stretch cap in the next morning. We recorded the 24-h drainage each day and removed the drainage device when this was < 10 mL for two consecutive days or after 8 days. A pressure dressing continued to bandage the operative region. All patients were under frequent observation and strict follow-up.

Drain fluid amylase (DFA) concentration, drainage volume and postoperative pressure time were recorded. A few days after the drainage tube was uprooted, the symptom was defined as salivary fistula if the patient developed an effusion in surgical region. In addition, the following data were collected for the analysis: basic characteristics of the patients, including sex and age; details of the surgical procedures, including the position and length of incision, intraoperative blood loss, protection of the greater auricular nerve and facial nerve, and the reconstruction of surgical defects; the incidence of permanent facial paralysis, Frey’s syndrome or postoperative numbness of the surgical region; the use of neurotrophic drugs; the pathologic diagnosis; and tumour recurrence.

## Analysis

Continuous variables are expressed as mean ± standard deviation or median (range). In the univariate analysis, patient characteristics and perioperative and postoperative factors were compared between groups using the chi-square test, Student’s t test, Fisher’s exact test or the nonparametric Mann–Whitney U test depending on the type of data and their distribution. Levene’s test was used to evaluate the equality of variances. Binary logistic regression analysis was applied, and receiver operating curve (ROC) analysis was used to estimate the optimal cut-off value (based on the Youden index) for the prediction parameter and to calculate the associated sensitivity and specificity for predicting salivary fistula development. *P*-values < 0.05 were considered statistically significant. The statistical analysis was performed with SPSS 22.0 software (IBM Corp., Armonk, NY, USA).

## Results

Of the 57 cases followed in this study, 35(61%) cases were male and 22 (29%)were female. The mean age of the patients was 50 ± 16 years (range, 14–81 years). All cases were postoperatively diagnosed as benign lesions in pathology. Pleomorphic adenoma (*n* = 23; 40%) and warthin tumour (*n* = 20; 35%) were the two common diagnoses. The remaining cases were diagnosed as branchial cyst (*n* = 4; 7%), basal cell adenoma(*n* = 3;5%), Kimura’s disease (*n* = 1; 2%), calcifying epithelioma (*n =* 1; 2%), cystadenoma (*n =* 1; 2%) and neurilemmoma (*n =* 1; 2%), while 3 cases were recorded as benign parotid tumour.

Excluding unrecorded data, intact preservation of the parotid–masseteric fascia was achieved in 17 cases; partial preservation was achieved in 35 cases, and the parotid–masseteric fascia was not preserved in 4 cases. There were 19 cases under tumour enucleation, while the rest of the cases were treated with parotidectomy. 15 cases with parotidectomy had no involvement of facial nerve dissection, 9 cases had branched facial nerve dissection and 12 cases had total facial nerve dissection. The demographic, intraoperative and postoperative characteristics of the patients with or without a salivary fistula are shown in Table 1. In the univariate analysis, surgical procedures, tumour pathology and the reconstruction of surgical defect showed no statistical association with postoperative salivary fistula development (*p* > 0.05; Fisher’s exact test). However, the extent of parotid–masseteric fascia persistence was found to be associated with salivary fistula development (*p* = 0.006; Fisher’s exact test), with a lower incidence of salivary fistulas in cases with an intact fascia than in cases with a partial fascia (*p* = 0.019; Fisher’s exact test). This suggested that keeping the parotid–masseteric fascia intact helped reduce salivary fistula morbidity.

**Table 1:** Demographic, intraoperative and postoperative characteristics

	**Ages (mean ± SD)**	***P*value**	
**Cases Without Salivary fistula**	**53 ± 17**	**0.098**	
**Cases Without Salivary fistula**	**45 ± 14**		
	**Case Without Salivary fistula**	**Cases With Salivary fistula**	***P*value**
**Gender(M/F)**	**24/15**	**11/7**	**0.975**
Surgical procedures^*^			
**a**	**13**	**6**	**0.618**
**b**	**8**	**7**	
**c**	**7**	**2**	
**d**	**9**	**3**	
**Pathology**			
**Pleomorphic adenoma**	**15**	**8**	**0.338**
**Warthin tumor**	**14**	**6**	
**Branchial cyst**	**4**	**0**	
**Kimura’s disease**	**1**	**0**	
**Calcifying epithelioma**	**1**	**0**	
**Basal cell adenoma**	**3**	**0**	
**Cystadenoma**	**0**	**1**	
**Neurilemmoma**	**0**	**1**	
**Parotid masseter fascia’s persistence**			
**Intact**			
**Partial**	**13**	**4**	0.006^**^
**Few**	**14**	**21**	
**Reconstruction of surgical defect**	**4**	**0**	
**none**			
**Sternocleidomastoid muscle flap**	**36**	**17**	
**Parotid flap**	**3**	**0**	**0.192**
	**0**	**1**	
	**Draining output (ml)** **(mean ± SD)**	**DFA(U/L)** **(median)**	**Duration of postoperative pressure dressingapplication (day)** **(mean ± SD)**
**Case Without** **Salivary fistula**	**58 ± 37.679**	**18,366.5**	**9.0 ± 4.485**
**Case With Salivary fistula**	**73 ± 60.042**	**80,132.5**	**9.3 ± 3.088**
***P value***	**0.283**	**< 0.001**	**0.818**

* Surgical procedure includes:a: tumor enucleation; b: tumor and partial superficial parotidectomy; c: tumor and partial superficial parotidectomy with branched facial nerve dissection; d: tumor and partial superficial parotidectomy with total facial nerve dissection

**P = 0.006 when comparing three extent of parotid masseter fascia’s persistence “intact”, “partial”, “few”; while P = 0.019 when comparing “intact” and “partial”

There was no statistical difference between salivary fistula and sex or age. Draining output volume showed no association with salivary fistula development (*p* = 0.283; Student’s t test) (Table 1). The postoperative pressure time(*p* = 0.015; Levene’s test) and DFA concentration(*p* = 0.001; Levene’s test) both showed heterogeneity of variance and were evaluated accordingly. The postoperative pressure time showed no association with salivary fistula development (*p* = 0.818; Mann–Whitney U test). However, DFA concentration showed a statistically significant association (*p* <  0.001; Mann–Whitney U test), with a significantly higher median concentration of DFA observed in the patients with a salivary fistula (18,400 vs. 80,100 U/L) (Table 2).

**Table 2:** Mean and Median value of drain fluid amylase and drainage volume

		**Volume in cases Without salivary fistula**	**Volume in cases With salivary fistula**
**Drain fluid amylase (U/L)**	**Range**	**27–261,136**	**34,215–716,823**
	**Mean**	**34,340.3**	**155,491.1**
	**Median**	**18,366.5**	**80,132.5**
**Drainage volume (ml)**	**Range**	**0–158**	**11–230**
	**Mean**	**58**	**73**
	**Median**	**57**	**51**

In a binary logistic regression model for salivary fistula development that included all the parameters, DFA concentration and the postoperative pressure time both showed statistical significance (DFA: β = 0.129, odds ratio [OR] for an increase of 1 KU/L in DFA concentration = 1.14, *p* = 0.035; postoperative pressure time: β = 1.367, OR = 3.923, *p* = 0.039; Table 3). This model was applied to predict the cases in this study that would develop salivary fistulas; the prediction was correct for 47 of the 53 cases (89%).

**Table 3:** Binary logistic regression model

	**β**	**P**	**OR**
Reconstruction of surgical defect (1) ^*^		1.000	
Reconstruction of surgical defect (2) ^*^	−19.794	0.999	0.000
Reconstruction of surgical defect (3) ^*^	24.441	1.000	41,176,069,965.360
Parotid masseter fascia’s persistence (1) ^*^		0.894	
Parotid masseter fascia’s persistence (2) ^*^	2.661	0.635	14.308
Parotid masseter fascia’s persistence (3) ^*^	−28.638	1.000	0.000
Surgical procedures (1) ^*^		0.478	
Surgical procedures (2) ^*^	−5.313	0.391	0.005
Surgical procedures (3) ^*^	−6.522	0.328	0.001
Surgical procedures (4) ^*^	1.588	0.786	4.895
Drainage volume	−0.011	0.714	0.989
Postoperative pressure time	1.367	0.039	3.923
DFA^**^	0.129	0.035	1.138
constant	−25.303	0.044	0.000

^*^: The Numbers in brackets represent dummy variables

^**^: The unit of DFA is KU/L = 1000 U/L

β: partial regression coefficient; OR: odds ratio; α = 0.05;

The model accuracy is 89%

**Fig. 1:**
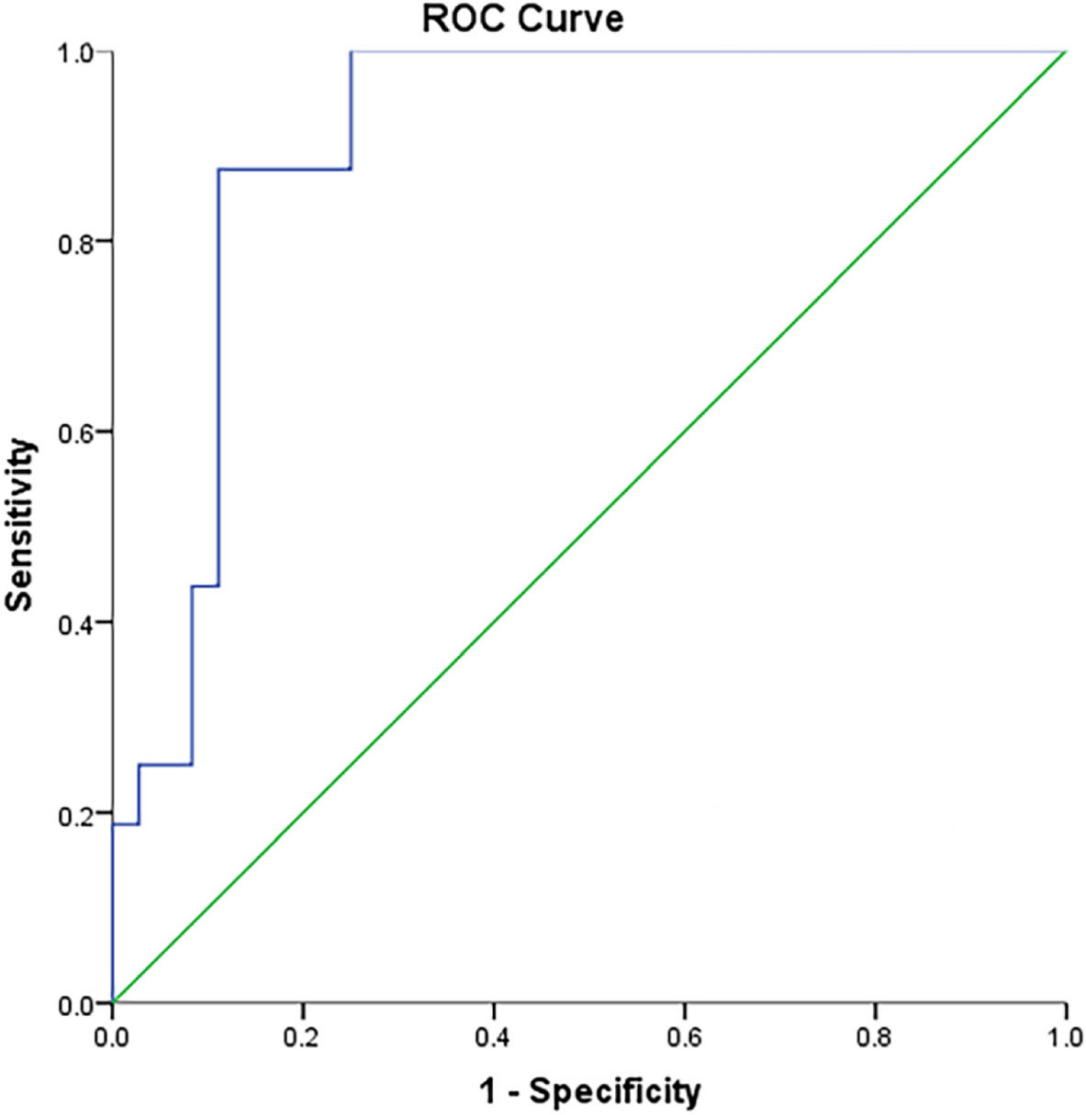
ROC (Receiver operating characteristics) curve of DFA and postoperative salivary fistula. AUC (Area Under Curve) is 0.903, which indicates that DFA volume has a good predictive value. Based on Youden index, the optimal threshold DFA level is 51,124.5 U/L, with a high sensitivity (0.875) and a high specificity (0.889).

We applied ROC analysis to further explore the predictive value of DFA concentration (Fig. 1). This showed a significant value for the area under the curve (0.903, *p* < 0.001), indicating that DFA concentration was a tenable diagnostic indicator. The optimal cut-off value for DFA concentration, determined by maximising Youden index, was 51,100 U/L; this gave a sensitivity of 0.875 and specificity of 0.889 for predicting salivary fistula development. Two cases that developed salivary fistula had DFA concentrations below the cut-off value (false negatives) and four cases without fistulas had concentrations above the cut-off value (false positives). A further cut-off value was established to avoid false negative results; with a cut-off value of 30,300 U/l, the sensitivity was 100% and specificity 75% (with no false negatives and 25% false positives).

## Discussion

Benign parotid tumours, which commonly occur in the superficial parotid lobe^2^, account for a large proportion of salivary neoplasms. Current surgical methods include enucleation and partial or complete superficial parotidectomy, but these are associated with complications such as salivary fistulas, weakness of the facial nerve and Frey’s syndrome, which cause functional and aesthetic discomfort to patients after surgery. In this study, we followed 57cases with the aim of establishing a predictive indicator for postoperative salivary fistula development. To eliminate distractions and obtain a more accurate conclusion, all the cases were diagnosed with benign neoplasms in the parotid, because whether the tumour is benign or malignant may affect the prognosis. Eighteen cases of salivary fistulas were identified.

A salivary fistula is generally considered to be the outcome of constant fluid secretion from saliva-producing parenchyma The overwhelming majority of fistulas develop within 1 month of surgery and classically occur during eating [4, 5]. The fistula may persist for a long time after complete wound healing**.** In this study, we observed an association between DFA concentration and postoperative salivary fistula development. The median DFA concentration was significantly higher in the fistula cases than in those without postoperative fistulas. A binary logistic regression model confirmed the positive relationship between DFA concentration and postoperative salivary fistula development, with an OR of 1.14 per increase of 1 KU/L in DFA concentration, and ROC analysis indicated that DFA concentration was a tenable diagnostic indicator, with an optimal cut-off DFA concentration of 51,100 U/L resulting in a sensitivity of 87% and specificity of 89%.

A salivary fistula is generally considered to be the outcome of constant fluid secretion from saliva-producing parenchyma. This relationship between DFA concentration and salivary fistula development was consistent with research by Larsen et al. [6] which showed an upward trend in DFA concentration with early fistulisation. DFA concentration and perioperative albumin ratio has been reported to be an effective predictor of pancreatic fistula development after pancreatomy [7–9], also allowing an assessment for the earlier removal of the drain [10]. We speculate that the reason for the association between DFA concentration and salivary fistula development may be as follows. Saliva leaking from parotid causes fluid retention due to the destruction of parotid tissue integrity, especially the non-closure of the main duct or interlobar ducts. The rise of amylase concentration may due to the damage of the parotid gland or duct, and this phenomenon could be more pronounced 3–5 days after surgery, as wound tissue fluid is decreased. Since the collection of drainage liquid is an event before removing drainage tube, it has predictive value in the judgement of salivary fistula.

In the course of previous treatments, we observed that a few cases with an extremely high DFA concentration and low draining output volume did not result in salivary fistula development. Hence, we examined whether the draining output volume was associated with fistula development. However, the present data showed no significant difference in median drainage volume between the groups with and without fistula development. We speculate that drain output is influenced by various factors. For example, Chen et al. [11] reported association between increased drain output and a high body weight or diabetes mellitus. In addition, women who are perimenopausal and postmenopausal have a lower saliva flow rate [12]; moreover, a strong association has been reported with gland size, which is related to body weight and body mass index [13].

We also considered whether there was a relationship with postoperative pressure time, but there was no significant difference in this parameter. Interestingly, the binary logistic regression model suggested a positive correlation between the postoperative pressure time and salivary fistula development, which was inconsistent with clinical experience. Most cases (8 of 9) in which the postoperative pressure time was less than 7 days did not result in salivary fistula development. We concluded that the postoperative pressure time applied to each case may have been affected by factors subjective to the clinicians. To relieve patients’ discomfort from pressure, postoperative pressure time was reduced insome cases where clinical experience suggested a low possibility of salivary fistula development. Conversely, pressure time was prolonged for cases that showed early signs of a salivary fistula to alleviate the symptoms. The postoperative pressure time, therefore, could not be used as a predictor of salivary fistula in this study.

The surgical procedure did not show statistically significant associations with fistula development. While the diversity has been reported in complications from different surgical procedures for benign tumour removal, with enucleoresection resulting in fewer cases of salivary fistula, temporary facial nerve weakness or facial paralysis compared with superficial or total parotidectomy [14].

Besides, reconstruction using a parotid fascia flap has been reported to help prevent postoperative fistula formation [15]. Consistent with this, cases in the present study with an intact parotid–masseteric fascia had a significantly lower fistula rate than those with a partially retained fascia (29% vs. 60%). In addition, use of the sternocleidomastoid muscle flap has been reported to be advantageous for reducing the onset of salivary fistula^16^ and Frey’s syndrome [16]. Superficial musculoaponeurotic system flap reconstruction can also play a role in the prevention of Frey’s syndrome and fistulas [14, 17, 18], providing isolation between postoperative facial nerve regeneration and interference from the parotid gland bed, with the addition of inhibition of parotid secretion accumulation. Although the superficial musculoaponeurotic system coheres firmly with the superficial aspect of the parotid fascia, they are separated by a deep fibroadipose connective layer [19].

## Conclusions

For patients with benign parotid mass, we suggest that DFA concentration has predictive value for identifying cases at risk of postoperative salivary fistula development. The incidence of salivary fistula development increases with DFA concentration, and the possibility of postoperative parotid fistula should be considered when the concentration exceeds the identified optimal cut-off value of 51,100 U/L. In addition, we recommend that the parotid–masseteric fascia should be preserved as completely as possible to reduce the incidence of salivary fistulas.

## Data Availability

The datasets used and analyzed during the current study are available from the corresponding author on reasonable request.

## Abbreviations

DFA: Drain fluid amylase
ROC: Receiver operating curve
OR: Odds ratio
