# Correction to: Drain fluid amylase as a predictor of postoperative salivary fistula in cases with benign parotid tumours

**DOI:** 10.1186/s12903-021-01656-3

**Published:** 2021-06-14

**Authors:** Yusheng Lu, Shijian Zhang, Canbang Peng, Wenyi Yang, Chenping Zhang, Zhenhu Ren

**Affiliations:** 1 Department of Oral and Maxillofacial-Head and Neck Oncology, Ninth People’s Hospital, School of Medicine, Shanghai Jiao Tong University, 639 Zhi-zao-ju Road, Shanghai, 200011 China; 2 Department of Oral and Maxillofacial Surgery (Zhang Zhiyuan Academician Workstation), Hainan Western Central Hospital (Shanghai Ninth People’s Hospital, Hainan Branch), Danzhou, 571700 Hainan China

## Correction to: BMC Oral Health (2020) 20:184 10.1186/s12903-020-01166-8

After publication of the original article [1], an error was identified in the authors’ affiliations list.

The incorrect authors’ affiliations list is:

1 Department of Oral and Maxillofacial Surgery (Zhang Zhiyuan Academician Workstation), Hainan Western Central Hospital (Shanghai Ninth People’s Hospital), Hainan Branch, Danzhou, Hainan 571700, China.

2 Department of Oral and Maxillofacial-Head and Neck Oncology, Ninth People’s Hospital, School of Medicine, Shanghai Jiao Tong University, 639 Zhi-zao-ju Road, Shanghai 200011, China.

The correct authors’ affiliations list is:

1 Department of Oral and Maxillofacial-Head and Neck Oncology, Ninth People's Hospital, School of Medicine, Shanghai Jiao Tong University, 639 Zhi-zao-ju Road, Shanghai, 200011, China.

2 Department of Oral and Maxillofacial Surgery (Zhang Zhiyuan Academician Workstation), Hainan Western Central Hospital (Shanghai Ninth People's Hospital, Hainan Branch), Danzhou, Hainan, 571700, China.

The original article has been corrected.

## References

[1] Lu et al. BMC Oral Health. 2020;20:184. 10.1186/s12903-020-01166-8.

